# Modification of the *Drosophila* model of *in vivo* Tau toxicity reveals protective phosphorylation by GSK3β

**DOI:** 10.1242/bio.20136692

**Published:** 2013-11-19

**Authors:** Giulia Povellato, Richard I. Tuxworth, Diane P. Hanger, Guy Tear

**Affiliations:** 1 MRC Centre for Developmental Neurobiology, King's College London, New Hunt's House, Guy's Hospital Campus, London SE1 1UL, UK; 2 School of Clinical and Experimental Medicine, University of Birmingham, The Medical School, Birmingham B15 2TT, UK; 3 Department of Neuroscience, King's College London, Institute of Psychiatry, De Crespigny Park, London SE5 8AF, UK

**Keywords:** Tau, Phosphorylation, GSK3β, *Drosophila*

## Abstract

Hyperphosphorylation of the microtubule associated protein, Tau, is the hallmark of a group of neurodegenerative disorders known as the tauopathies which includes Alzheimer's disease. Precisely how and why Tau phosphorylation is increased in disease is not fully understood, nor how individual sites modify Tau function. Several groups have used the *Drosophila* visual system as an *in vivo* model to examine how the toxicity of Tau varies with phosphorylation status. This system relies on overexpression of Tau from transgenes but is susceptible to position effects altering expression and activity of the transgenes. We have refined the system by eliminating position effects through the use of site-specific integration. By standardising Tau expression levels we have been able to compare directly the toxicity of different isoforms of Tau and Tau point mutants that abolish important phosphorylation events. We have also examined the importance of human kinases in modulating Tau toxicity *in vivo*. We were able to confirm that human GSK3β phosphorylates Tau and increases toxicity but, unexpectedly, we identified that preventing phosphorylation of Ser404 is a protective event. When phosphorylation at this site is prevented, Tau toxicity in the *Drosophila* visual system is increased in the presence of GSK3β. Our data suggest that not all phosphorylation events on Tau are associated with toxicity.

## Introduction

The tauopathies are a group of neurodegenerative diseases characterised by the accumulation of intra-neural aggregates of the microtubule-associated protein, Tau. They include Alzheimer's disease (AD), fronto-temporal lobar degeneration (FTLD-Tau), Pick's disease, progressive supranuclear palsy and corticobasal degeneration. The microtubule-binding properties of Tau were identified more than 30 years ago but, and despite intense research, its true physiological role remains unclear, thus the cellular mechanisms underpinning the tauopathies are also poorly understood. More recently, additional properties of Tau have been identified, including an ability to bundle actin filaments, and multiple Tau-binding partners have been identified, including tyrosine kinases. Together, these have led to the suggestion that Tau may act as a signalling scaffold (reviewed by Morris et al., 2011).

The functions of Tau may be regulated both by alternative splicing (six isoforms are expressed in the human CNS) and through a multitude of post-translational modifications. These include multiple phosphorylation and acetylation events, glycosylation, ubiquitylation and sumolyation amongst others (reviewed by Martin et al., 2011). The phosphorylation of Tau has received the most attention because Tau within neuropathological aggregates is found primarily in a highly phosphorylated form (Buée et al., 2000; Lee et al., 2001; Ballatore et al., 2007; Hanger et al., 2009; Iqbal et al., 2009). *In vitro*, hyperphosphorylation of Tau reduces its microtubule binding affinity (Lindwall and Cole, 1984). This leads to an increase of hyperphosphorylated Tau in the cytosol and a destabilization of the microtubule network (Cowan et al., 2010; Feuillette et al., 2010). Both the accumulation of highly phosphorylated cytosolic Tau and destabilization of the microtubules are suggested to lead to neurodegeneration (Cowan et al., 2010; Wu et al., 2011). Yet, increased phosphorylation of Tau is not necessarily toxic to neurons in all circumstances as it occurs during fetal development (Yu et al., 2009) and transiently in hibernating mammals (Arendt et al., 2003). An alternative view sees elevated Tau phosphorylation as part of a protective response to oxidative stress rather than a direct cause of pathology (Castellani et al., 2008; Bonda et al., 2011). The complex relationship between Tau phosphorylation and neurodegeneration is also highlighted by observations that phosphorylation resistant Tau constructs either lose or maintain their toxicity, suggesting a non-equivalent role for groups of phosphorylation sites. In *Drosophila* both TauAP and TauS11A forms, with 14 or 11 Ser/Thr sites mutated to alanine respectively, retain microtubule binding function yet TauAP loses toxicity while TauS11A retains toxicity (Steinhilb et al., 2007b; Feuillette et al., 2010; Talmat-Amar et al., 2011). Other studies have shown alternatively that phosophorylation of Tau at specific sites can promote microtubule binding or reduce Tau toxicity (Wada et al., 1998; Feijoo et al., 2005; Thies and Mandelkow, 2007) while increased binding of Tau to microtubules may also be deleterious to neurons through interference with axonal trafficking (Talmat-Amar et al., 2011). Thus, a precise balance of differential Tau phosphorylation at individual sites may be required to appropriately regulate levels of cytosolic or microtubule bound Tau essential for microtubule dynamics and axon transport.

Since Tau phosphorylation is likely to contribute in some way to pathology, one therapeutic strategy being followed is to reduce the phosphorylation load on Tau by targeting Tau kinases (Churcher, 2006; Mazanetz and Fischer, 2007; Brunden et al., 2009). For this approach to be effective, it is important to identify which of the many Tau phosphorylation events that have been identified *in vitro* are critical for toxicity *in vivo* and to establish which kinases phosphorylate Tau in disease states and whether Tau forms resistant to phosphorylation show reduced toxicity. The scale of this task is significant because recent studies have identified 45 distinct sites that are phosphorylated on Tau from AD brains compared with only 17 from healthy brains, with many different kinases capable of phosphorylating Tau *in vitro* (Morishima-Kawashima et al., 1995; Hanger et al., 1998; Hanger et al., 2007; Hanger et al., 2009; Lebouvier et al., 2009). Moreover, some phosphorylation events will modify Tau *in vitro* but not necessarily in a manner that is physiologically relevant. There is a need, therefore, for a model system in which individual kinases can be tested for their ability to alter Tau toxicity *in vivo*.

Tau-mediated degeneration of photoreceptor neurons in the *Drosophila* visual system is a commonly used *in vivo* model to study the cell biology of the tauopathies (Wittmann et al., 2001; Jackson et al., 2002; Muqit and Feany, 2002; Nishimura et al., 2004; Karsten et al., 2006; Steinhilb et al., 2007a; Khurana, 2008; Chatterjee et al., 2009). Typically, human Tau is expressed ectopically in the developing *Drosophila* brain or visual system, resulting in neurodegeneration that bears several hallmarks of the tauopathies, including age dependency, abnormally phosphorylated Tau and, in some cases, Tau aggregates (e.g. Wittmann et al., 2001; Jackson et al., 2002; reviewed by Gistelinck et al., 2012). The powerful genetics of *Drosophila* can be employed to identify endogenous genes that are required for tau-mediated degeneration or can modify the degree of degeneration mediated by human Tau. Using this approach, several *Drosophila* kinases, including the homologues of GSK3β, MARK, cdk5, JNK and PKA, have been implicated in Tau toxicity (Wittmann et al., 2001; Jackson et al., 2002; Shulman and Feany, 2003; Chau et al., 2006; Steinhilb et al., 2007a; Steinhilb et al., 2007b; Chatterjee et al., 2009).

Following our studies of Tau phosphorylation in AD post-mortem brain (Hanger et al., 1998; Hanger et al., 2007), we were interested to determine whether the *Drosophila* photoreceptor model could be used to assess the roles of human kinases in mediating neurodegeneration *in vivo* and to identify particular phosphorylation events on Tau that are important for toxicity. Transgene expression in *Drosophila* is affected by positional effects on transgene activity which complicate comparisons of the toxicity mediated by different isoforms or mutant forms of human Tau. To overcome this, we used an alternative methodology to express Tau where Tau transgenes are targeted to pre-determined sites in the *Drosophila* genome to control for any positional effects and permit direct comparisons of toxicity. We sought to confirm the importance of GSK3β as a pathological kinase *in vivo*, both individually and in combination with CK1δ or DYRK1A, both of which could act as priming kinases for GSK3β by phosphorylating Tau residues adjacent to GSK3β target sites (reviewed by Doble and Woodgett, 2003). CK1δ is highly overexpressed in AD brain (Ghoshal et al., 1999) and is tightly associated with PHF Tau (Kuret et al., 1997). DYRK1A is both overexpressed in AD brain material and duplicated in the critical region of chromosome 21 in Down's syndrome which is associated with early onset AD (Kimura et al., 2007). We were able to confirm the importance of GSK3β for toxicity *in vivo*, but found little role for priming kinases in this system. Surprisingly, we identified a GSK3β-mediated phosphorylation event that seems to be protective. We also found that, in contrast to previous work, the R406W mutation in Tau associated with FTLD-Tau has no effect on toxicity in this system.

## Results

### Tau-mediated degeneration in transgenic flies is modified by position effects

**Fig. 1.**
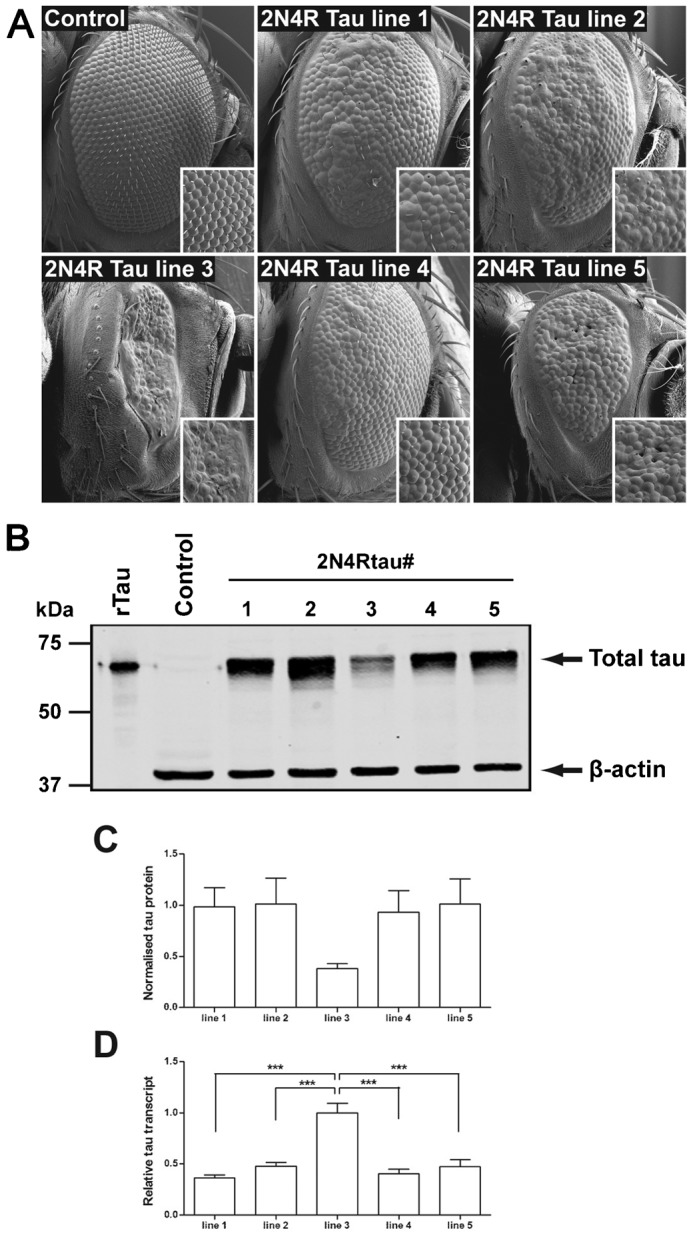
Position effect causes variations in Tau-mediated toxicity in the *Drosophila* eye. (A) Scanning electron micrographs of *Drosophila* eyes. The regular array of ommatidia of the compound eye is disrupted by overexpression of Tau to a variable degree. The GMR-gal4 driver was mated to wild-type (control) or UAS-2N4R Tau flies. Five different randomly inserted UAS-lines were assayed. (B, C) Quantitative Western blotting of Tau levels in adult *Drosophila* heads from GMR-gal4 × UAS-2N4R Tau flies. B. Blot probed with anti-total Tau and anti-β-actin. (C) Quantification of Tau protein levels normalised to actin. Recombinant Tau was included to permit normalisation between blots. (D) Tau transcript levels in brain and eye discs from third instar larvae before degeneration occurs. Tau transcripts were normalised to membrane GFP expressed from the GMR-gal4 chromosome after recombination (see Materials and Methods).

We sought to use *Drosophila* as a model to assess the roles of human kinases to generate toxic forms of Tau and to identify the particular phosphorylation events on Tau responsible for toxicity. Previous studies of human Tau toxicity in *Drosophila* have used transgenic lines generated by P-element mediated transgenesis (Wittmann et al., 2001; Jackson et al., 2002; Nishimura et al., 2004; Steinhilb et al., 2007a; Steinhilb et al., 2007b; Chatterjee et al., 2009) where constructs containing human Tau cDNA are inserted at random into the *Drosophila* genome (Spradling and Rubin, 1982). Expression of Tau is achieved using the UAS/GAL4 system or by use of a tissue specific promotor. However variable levels of expression results from the same Tau construct inserted at different genomic locations. This position-dependent expression is a well-characterised phenomenon of P-element mediated transgenesis in *Drosophila* and, whilst this can be exploited to obtain a range of expression levels (Wilson et al., 1990), it may limit the ability to directly compare the toxicity of variant forms of the transgene. We investigated the extent to which Tau toxicity can vary due to positional effects resulting from random insertion of the same transgene. We generated five different random insertions of UAS-2N4R Tau and used GMR-GAL4 to drive Tau expression in the developing visual system from one copy of the transgene. The disruption of the regular, crystalline array of ommatidia in the adult compound eye ranged from mild (e.g. line 4) to severe (e.g. line 3) (Fig. 1A).

We identified the genomic insertion site of UAS-Tau for each line (supplementary material Table S1). Four of the five lines (lines 1, 2, 4, and 5) were homozygous viable insertions and none of these showed an eye phenotype in the absence of the GMR-GAL4 driver. Line 3 exhibited the most severe degeneration (Fig. 1). This line contained Tau inserted into the l(3)87Df gene, and was lethal when homozygous. To exclude the possibility that the severe degeneration apparent in line 3 was due to a genetic interaction between Tau and l(3)87Df gene, which encodes a probable chaperone, we used GMR-GAL4 to express Tau from the mildly disruptive line 1 locus in a genetic background heterozygous for an allele of l(3)87Df. No increase in degeneration resulted, excluding the possibility that l(3)87Df plays a role in Tau-mediated degeneration (supplementary material Fig. S1). We concluded that the Tau-mediated degeneration is highly dependent on positional effects and we would need to control for these if we were going to compare the toxicity of different forms of Tau.

### The amount of Tau protein does not correlate with degeneration

To determine the relationship between the level of disruption to the eye in the differing transgenic lines and Tau protein expression, we quantified the amount of Tau expressed in each line in *Drosophila* heads normalised to β-actin (Fig. 1B,C). We detected significantly less Tau protein in line 3, compared to the other lines, despite this line displaying the most severe degeneration. The amounts of Tau protein present in lines 1, 2, 4, and 5 were equivalent to each other, despite differences in the degree of disruption to the eye (Fig. 1C). The reduced level of Tau protein in the lines with greater levels of disruption may potentially be due to increased numbers of dying or dead cells. To circumvent this we used qPCR to measure transcript levels of each Tau transgene earlier in the development of the visual system, within late third instar larval eye imaginal discs, prior to onset of degeneration. We controlled for the number of cells expressing GMR-GAL4 (and therefore Tau) by recombining UAS-GFP with GMR-Gal4 to drive the expression of GFP. Normalisation of the Tau transcript levels to that of GFP for each line revealed significantly increased Tau transcription in the highly degenerate line 3 compared to all of the other Tau insertion lines (Fig. 1D). Lines 1, 2, 4, and 5 each express approximately equivalent amounts of Tau mRNA and protein, despite variation in the levels of Tau toxicity apparent in each line. Line 3 expresses significantly more Tau mRNA which correlates with the more severe degenerate phenotype, suggesting the lower levels of Tau protein in adults is a consequence of increased cell loss. Thus, the level of toxicity mediated by 2N4R-Tau driven from differing transgenes varies significantly and is influenced by positional effects. We could not be confident that protein or mRNA levels would predict toxicity sufficiently accurately to be used as a reliable method for selecting comparable insertion lines.

### Site-specific integration of transgenes eliminates the effects of positional variation

**Fig. 2.**
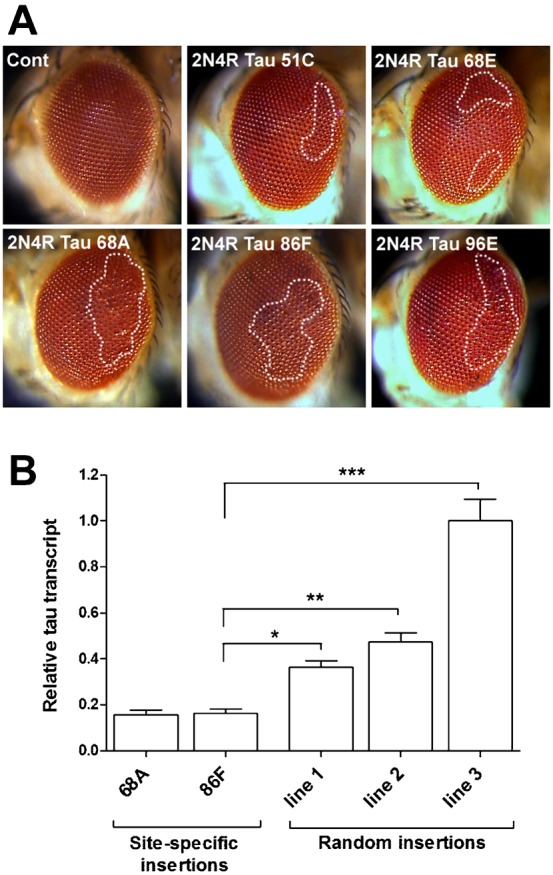
Site-specific insertions of UAS-2N4R Tau mediate lower levels of toxicity. UAS-2N4R Tau was inserted into five specific genomic landing sites by φC31-mediated integration. (A) Light micrographs of adult eyes from GMR-gal4 × wild-type (control) or UAS-2N4R Tau crosses. Areas of the eye displaying significant disruption are highlighted with dashed lines. (B) Tau transcript levels driven by GMR-gal4 from two site-specific UAS insertions normalised to our random insertion line 3.

To overcome the problem of positional effects on Tau expression, we turned to the φC31 system that allows site specific integration of *Drosophila* transgenes at specific “landing” sites in the genome (Groth et al., 2004). We integrated UAS-2N4R Tau at five different landing sites spread across chromosomes II and III (insertion sites at 51C, 68A, 68C, 86F and 96E). After crossing to GMR-GAL4 driver flies, toxicity in the eyes was assessed (Fig. 2A). In two cases (51C and 68E; Fig. 2A, upper row) there was very little disruption of the crystalline array of ommatidia; in three others (68A, 86F and 96E; Fig. 2A, lower row) disruption was moderate. In none of the lines was disruption seen to the level of any of our randomly integrated lines (cf. Fig. 1). We quantified Tau transcript expression in eye discs from two of the five site-specific lines, 68A and 86F, relative to GFP, as before. As expected, Tau is expressed at significantly lower levels in both of these lines. Tau expression levels in the targeted insertion lines was approximately half of the level in the random insertion lines 1 and 2 and considerably lower than the highly degenerate line 3 (Fig. 2B). To check reproducibility of the landing sites, we generated an additional independent line with UAS-2N4R Tau inserted at the 68A locus. This second insertion generated an identical eye phenotype to the initial 68A transgenic when expression was driven with GMR-GAL4 (data not shown).

### Using site-specific insertion lines, R406W mutation does not enhance the toxicity of Tau

**Fig. 3.**
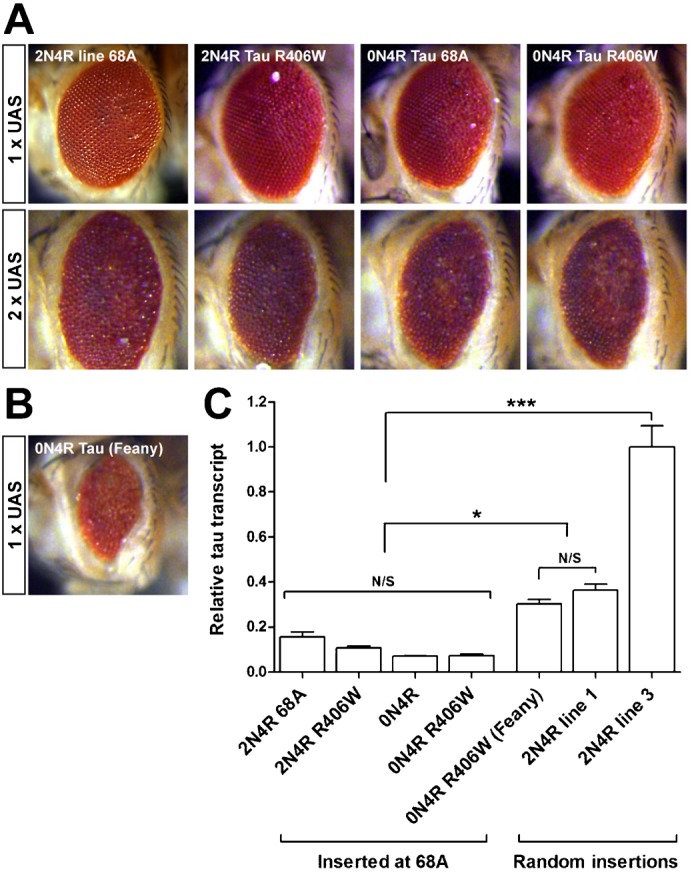
No increase in Tau-mediated toxicity caused by the R406W mutation. (A) GMR-gal4 driving expression from the 68A integration site of 2N4R or 0N4R Tau with or without the R406W mutations. Upper row: one copy of the UAS-insert. Lower row: two copies of the UAS-insert. GMR-gal4 is heterozygote in all cases. The R406W mutation does not increase toxicity. (B) GMR-gal4 driving 0N4R Tau R406W from a randomly inserted UAS-line generated by the Feany lab (Wittmann et al., 2001). (C) Tau transcripts levels driven by GMR-gal4 normalised to our random insertion line 3.

We used the φC31-mediated site-specific insertion to test the contribution of specific residues within Tau to cause toxicity *in vivo*. The R406W mutation in Tau is associated with some cases of autosomal dominant FTLD-Tau (Hutton et al., 1998) and has been reported to be more toxic than wild-type Tau when overexpressed in *Drosophila* (Wittmann et al., 2001; Jackson et al., 2002; Khurana et al., 2006). The R406W mutation affects the ability of Tau to regulate microtubule dynamics *in vitro* (Bunker et al., 2006) and impairs axonal transport in mice when overexpressed at high levels (Zhang et al., 2004) and may also affect the phosphorylation of Tau by specific kinases (Mack et al., 2001). We used GMR-GAL4 to drive expression of the 2N4R and 0N4R wild-type and R406W Tau isoforms from constructs inserted into the 68A locus. We found that there were no differences in toxicity between unmodified 0N4R or 2N4R Tau and those containing the R406W point mutant when expressed from the same insertion site, in contrast to the severe increase in toxicity in the eye reported previously for the R406W mutation (Wittmann et al., 2001; Jackson et al., 2002; Nishimura et al., 2004) (Fig. 3A, upper panels). In parallel, we examined the eye phenotype of flies overexpressing 0N4R Tau R406W from the randomly inserted construct generated by the Feany lab and used in several previous studies (Wittmann et al., 2001; Jackson et al., 2002; Nishimura et al., 2004) and confirmed this line generated a much stronger phenotype in the eye (Fig. 3B). We quantified transcript expression in larval eye discs and normalised expression to that of the strongest 2N4R Tau random insertion, line no. 3 (Fig. 3C). This revealed that each of the four Tau isoforms were expressed from the 68A site at similar levels, as expected (no significant differences were observed; Fig. 3C), but notably, expression from the 68A insertion site was approximately half that from four of the five randomly integrated lines we generated. The 0N4R Tau R406W line was expressed at a level twice that of the lines inserted at 68A and comparable to the level seen for most of our random insertions (Fig. 3C). We wondered whether Tau expression from the 68A insertions was below a threshold needed to observe any differential toxicity. We increased expression by combining GMR-GAL4 with double the copy number of the 68A UAS-Tau insertion in homozygous animals. As expected, the eye phenotype worsened in each case (Fig. 3A, lower panels) but the R406W variants of both 2N4R and 0N4R Tau continued to show similar toxicity to the non-mutated forms.

### Assay of human GSK3β activity for Tau toxicity

The low level of Tau overexpression from our 68A insertions results in moderate toxicity and provides a baseline to investigate factors that increase toxicity. This level of expression may be representative of human disease where Tau expression is not elevated.

**Fig. 4.**
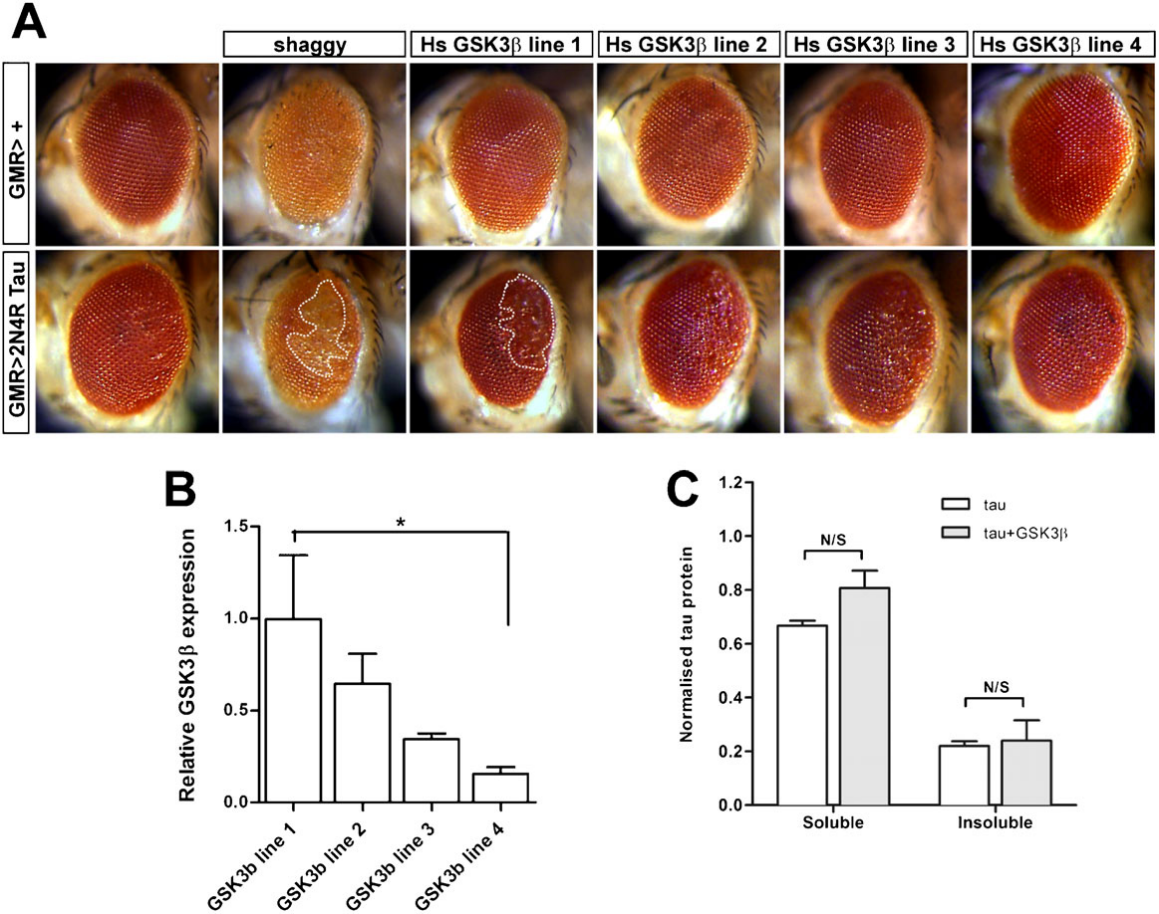
Co-expression of human GSK3β increases Tau-mediated toxicity. (A) Light micrographs of adult fly eyes. *Drosophila* (Shaggy) or hGSK3β was driven in the visual system under the control of GMR-gal4 either alone (upper row) or alongside 2N4R Tau from the 68A insertion site. Shaggy expression alone causes disruption of eye development but hGSK3β does not. Co-expression of GSK3β increases the toxicity of Tau. Areas of the eye displaying significant disruption including substantial areas of glazing are highlighted with dashed lines. (B) Relative expression of hGSK3β driven from four random UAS-insertions. Higher transcript levels correspond to higher Tau-mediated toxicity. (C) Increased toxicity does not correlate with a change in solubility of Tau. Tau in sarcosyl-soluble and -insoluble fractions was quantified by western blotting with or without co-expression of hGSK3β.

Several groups have previously investigated phosphorylation of exogenous human Tau by endogenous *Drosophila* kinases (Nishimura et al., 2004; Steinhilb et al., 2007a; Steinhilb et al., 2007b). A key role has been reported for Shaggy, the *Drosophila* homologue of GSK3β, consistent with the suggestion that GSK3β is a major pathological kinase in human tauopathies (Hanger et al., 1992; Lovestone et al., 1994; Lucas et al., 2001; Huang and Klein, 2006). We investigated whether Shaggy overexpression could similarly increase Tau toxicity in our targeted expression system. We used the GMR-GAL4 driver to express Shaggy from a UAS-*shaggy* transgene in the presence or absence of 2N4R Tau inserted at the 68A locus. We found that, whereas expression of Shaggy alone caused mild disruption of the ommatidia, co-expression with 2N4R Tau resulted in increased eye disruption (Fig. 4A, dashed area highlights severe disruption and glazing of the eye). The ommatida were more severely disrupted and patches of pigment loss and glazing were visible in the eyes of the Shaggy and Tau expressing flies. Thus, Tau-mediated toxicity can be increased by co-expression of human Tau with *Drosophila* GSK3β, even at low levels of Tau expression.

In order to examine whether human GSK3β (hGSK3β) could also enhance Tau-mediated toxicity in *Drosophila*, we first confirmed that hGSK3β was capable of phosphorylating human Tau in *Drosophila* cells. When hGSK3β was co-expressed with human 2N4R Tau in *Drosophila* S2 cells, we identified hGSK3β-mediated phosphorylation events using PHF1, AT8, AT270 and AT100 antibodies which recognise specific Tau phospho-epitopes (supplementary material Fig. S2). We next generated four UAS-hGSK3β transgenic lines by random insertion and found each line was expressed to varying amounts (Fig. 4B) yet expression of hGSK3β alone did not disrupt the ommatidial structure (Fig. 4A, upper panels). When hGSK3β expression was driven together with 2N4R Tau inserted in the 68A locus an enhanced disruption of the eye occurred (Fig. 4A, lower panels). The degree of enhancement was dependent on the level of GSK3β expression from each transgene (Fig. 4A,B). hGSK3β line 1 has the highest expression and generated the greatest toxicity when expressed with Tau, all subsequent experiments were performed using this hGSK3β line 1. We examined whether the increased Tau toxicity caused by hGSK3β was a consequence of changes to Tau solubility since altered solubility of Tau is associated with neurodegenerative tauopathies (Lewis et al., 2000; Ishihara et al., 2001; Zhukareva et al., 2002; Hirata-Fukae et al., 2009). Sarcosyl fractionation is a commonly used method to assay Tau solubility (Lewis et al., 2000; Zhukareva et al., 2002). We extracted sarcosyl-soluble and sarcosyl-insoluble fractions from *Drosophila* heads and found no change in the ratio of soluble to insoluble Tau in these fractions when hGSK3β was co-expressed together with Tau in *Drosophila* (Fig. 4C).

### GSK3β-mediated phosphorylation events on Tau

**Fig. 5.**
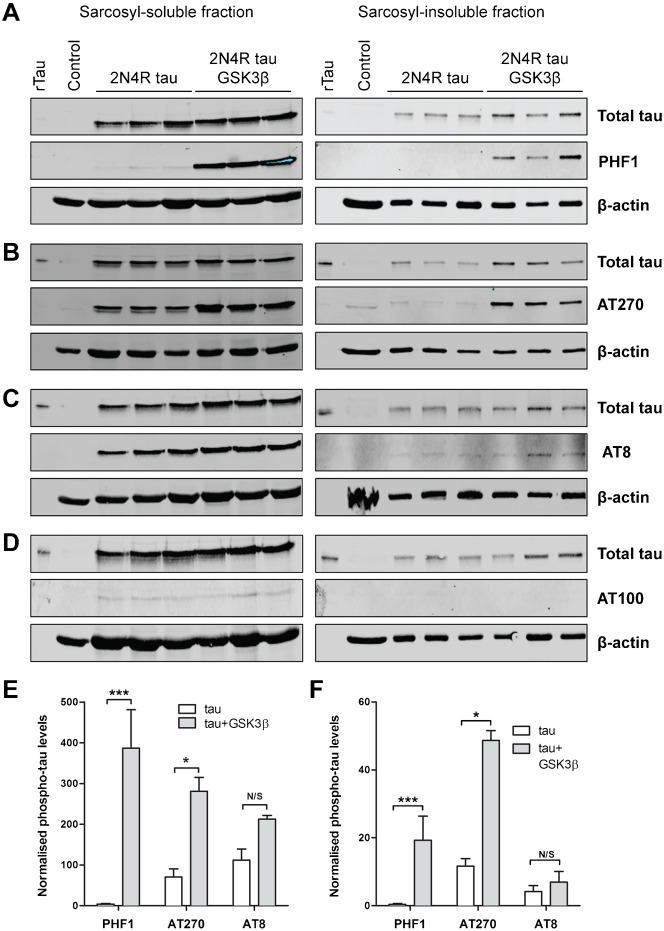
Tau phospho-epitopes phosphorylated by human GSK3β in the *Drosophila* visual system. Sarcosyl-soluble and -insoluble Tau fractions were purified from fly heads overexpressing 2N4R Tau from the 68A locus with or without hGSK3β. Four phospho-specific antibodies were used to identify specific phosphorylation events mediated by hGSK3β *in vitro*.

We used antibodies to four phospho-epitopes, in addition to total Tau to examine the effects of hGSK3β expression on phosphorylation of Sarcosyl-soluble and Sarcosyl-insoluble extracts in the Tau transgenic flies. Three of the antibodies (AT270, PHF1, and AT8) recognise known GSK3β target sites in Tau (Thr181, Ser396/404, and Ser202/Thr205, respectively), whereas labelling by antibody AT100 requires phosphorylation of both Thr212 and Ser214 by GSK3β in combination with another kinase (Sato et al., 2002). Soluble and insoluble Tau was readily detected in the heads from *Drosophila* expressing the 2N4RTau transgene inserted into the 68A landing site (Fig. 5A–D). We found at the levels of Tau expression seen in our system neither soluble or insoluble Tau were phosphorylated appreciably on Ser396/Ser404 by endogenous *Drosophila* kinases but both fractions were phosphorylated when hGSK3β was co-expressed (Fig. 5A,E,F; PHF1). Residue Thr181 on Tau was phosphorylated in both soluble and insoluble fractions by endogenous kinases and this phosphorylation was significantly increased by hGSK3β (Fig. 5B,E,F; AT270). In contrast, Ser202/Thr205 was phosphorylated by endogenous kinases but phosphorylation at this epitope was not increased by hGSK3β (Fig. 5C,E,F; AT8). Phosphorylation of Thr212 and Ser214 was barely detectable in soluble Tau and was absent from insoluble Tau. Furthermore, this epitope was not generated by co-expression of hGSK3β (Fig. 5D; AT100). Thus, Tau residues Ser202/Thr205 and Thr212/Ser214 are not targets for hGSK3β *in vivo*, at least in *Drosophila*, but phosphorylation of Thr231 and Ser396/Ser404 is substantially increased in this system.

### No role for GSK3-priming kinases in mediating Tau toxicity in the *Drosophila* eye

Our failure to detect phosphorylation of Thr212/Ser214 by hGSK3β to generate the AT100 epitope may indicate a requirement for priming at adjacent upstream Ser residues by an additional kinase (Doble and Woodgett, 2003). One candidate is casein kinase 1δ (CK1δ) which acts co-operatively withGSK3β on some substrates (Zeng et al., 2005). CK1δ is highly overexpressed in AD brain (Ghoshal et al., 1999) and our previous work has indicated a potential role for this kinase in aberrant phosphorylation of Tau (Hanger et al., 2007). We also wished to see if dual-specificity tyrosine phosphorylation regulated kinase 1A (DYRK1A) would act similarly. DYRK1A phosphorylates Tau *in vitro* and acts as a priming kinase for GSK3β (Woods et al., 2001). The DYRK1A gene lies within the critical region on chromosome 21 and is duplicated and overexpressed in Down Syndrome (DS). DS patients have a high incidence of early onset AD and one possible cause is hyperphosphorylation of Tau by the overexpressed DYRK1A (Ryoo et al., 2007; Liu et al., 2008). We generated transgenic UAS-human CK1δ and UAS-rat DYRK1A lines by random insertion. CK1δ was truncated after amino acid 371 because the shorter form is constitutively active. The kinases were expressed alongside Tau either with or without hGSK3β but the toxicity was not enhanced in any situation (supplementary material Fig. S3).

### GSK3β-mediated S404 phosphorylation is protective

**Fig. 6.**
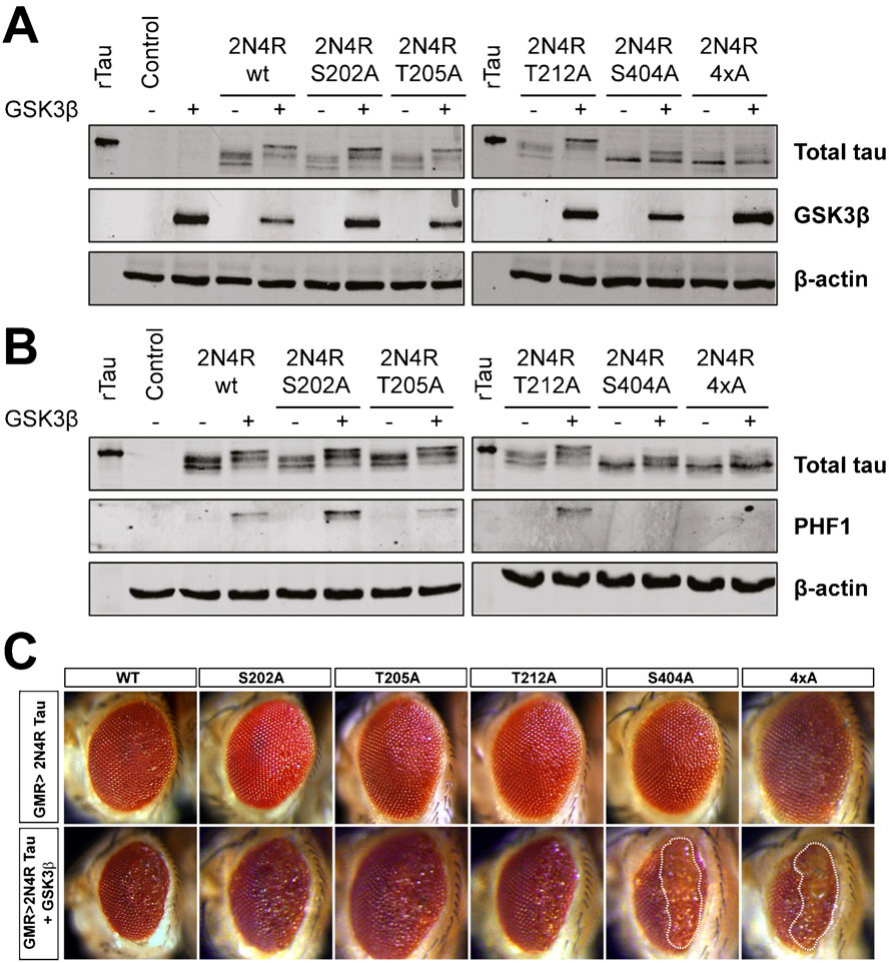
Phosphorylation of S404 by human GSK3β is protective in the *Drosophila* visual system. (A,B) Wild-type or point mutated forms of human 2N4R Tau were expressed in *Drosophila* S2 cells with or without myc-tagged hGSK3β. (A) Multiple bands representing differentially phosphorylated forms of Tau were detected between 60 and 70 kDa with a phospho-independent antibody. Co-expression of hGSK3β retards Tau mobility, indicative of increased phosphorylation load but this is absent in the Tau S404A or 4xA mutants. Recombinant Tau was included as a positive control for anti-Tau. (B) The pS396/pS404 epitope recognised by PHF1 is phosphorylated by hGSK3β but not by endogenous kinases. Phosphorylation is absent in the Tau S404A and the 4xA mutants. (C) Light photomicrographs of adult fly eyes. GMR-gal4 was used to express wild-type 2N4R Tau or S202A, T205A, T212A or S404A or Tau4xA Tau point mutants (top row). hGSK3β was co-expressed with each Tau isoform (bottom row). Areas displaying severe disruption to eye development including glazing and loss of pigmentation are highlighted by dashed lines. The S404A and 4xA mutant display increased toxicity when co-expressed with hGSK3β.

To determine which, if any, of the hGSK3β sites on Tau that we confirmed as phosphorylated *in vivo* are essential for increased toxicity, we replaced the individual residues, Ser202, Ser205, Thr212 or Ser404 in 2N4R Tau with non-phosphorylatable alanine. In a fifth mutant, all four of these residues were substituted by alanine (termed Tau4xA). We examined the effect of these mutations on the ability of hGSK3β to phosphorylate 2N4R Tau in *Drosophila* cells. When 2N4R Tau is expressed in cells, multiple Tau bands were detected using a phospho-independent antibody. These likely correspond to different phosphorylated forms mediated by endogenous kinases. Co-expression of hGSK3β resulted in a retardation of the Tau bands in the gel, indicating increased phosphorylation (Fig. 6A; see 2N4R wt). Mutating Tau at S202, S205 or T212 had no effect on the banding pattern of Tau, either with or without co-expression of hGSK3β (Fig. 6A). In contrast, introducing the S404A mutation abolished the slowest migrating Tau bands, suggesting that this site is an important target of hGSK3β. The 4xA form of Tau carrying each of the four point mutations showed an identical reduction in phosphorylation and, as expected, the PHF1 antibody, which recognises the pS396/pS404 epitope on Tau, detected phosphorylation by hGSK3β on WT, S202A, S205A and T212A forms of Tau not in the S404A and 4xA mutant forms (Fig. 6B).

We made transgenic flies by inserting the mutant Tau constructs into the same 68A locus as before to examine the resistance of each of these mutations to hGSK3β-induced toxicity. When each of the single point mutations, Tau4xA, or wild-type Tau, was expressed in the *Drosophila* visual system, toxicity was equivalent. Each resulted in mild disruption of ommatidia (Fig. 6C, also cf. Fig. 2). Co-expression of three of the Tau mutants, Tau S202A, T205A or T212A, with hGSK3β, resulted in no changes in toxicity (Fig. 6). However, hGSK3β expression unexpectedly increased the amount of degeneration observed with either TauS404A or Tau4xA. The eyes had a greater degree of external disorganisation and extensive patches of depigmentation indicative of increased toxicity (Fig. 6; depigmented areas marked by dashed lines). This result indicates that phosphorylation of Ser404 in Tau by hGSK3β modulates the toxicity of Tau *in vivo* but in this system, phosphorylation may not increase Tau toxicity but instead may be protective against degeneration.

## Discussion

Hyperphosphorylated Tau is associated with a number of neurodegenerative diseases, strongly suggesting that disregulated phosphorylation of Tau leads to neural pathology. In order to progress towards effective therapies it is key to identify which of the many Tau phosphorylation events are critical for toxicity *in vivo* and to establish which kinases phosphorylate Tau to cause disease. Previous studies have used *Drosophila* to identify endogenous kinases that increase Tau toxicity (Jackson et al., 2002; Mudher et al., 2004; Nishimura et al., 2004; Chatterjee et al., 2009). We were interested to determine whether the *Drosophila* photoreceptor model could be used to assess the roles of human kinases in mediating neurodegeneration *in vivo* and to identify particular phosphorylation events on Tau that are important for toxicity. Use of this model requires that the individual transgenic lines generated to test manipulated forms of Tau be expressed identically. However, we and others have demonstrated that Tau transgenes randomly integrated within the *Drosophila* genome produced variable levels of expression. This presented a significant challenge in seeking to identify the contribution to *in vivo* toxicity of Tau phosphorylation events mediated by human kinases. In previous studies, transgenic flies were selected for comparable Tau expression based on western blot analysis (Wittmann et al., 2001; Steinhilb et al., 2007a; Steinhilb et al., 2007b). However, we have found a highly degenerate eye phenotype was associated with reduced Tau protein, presumably due to cell death. Rather, the toxicity correlated with the level of RNA expression at earlier developmental stages, before cell death was apparent. We demonstrated that the toxicity mediated by different forms of Tau was difficult or impossible to compare even after selecting lines with comparable protein or mRNA expression levels because even small changes in Tau transcript level resulted in quite variable degrees of toxicity. We concluded that, using the standard method of generating transgenic flies that is susceptible to position effects, it would be difficult to be certain that changes in toxicity mediated by different forms of Tau were due to the properties of the Tau protein itself rather than subtle changes in expression level.

### Controlling for integration site is necessary to assess Tau toxicity

To remove any possible confounding positional effects generated by random integration of Tau transgenes, we adopted φC31-mediated site-specific integration to standardise the genomic integration locus (Groth et al., 2004). We found that, as expected, Tau transgenes inserted into the same locus resulted in the equivalent expression of tau transcript. We selected *Drosophila* landing sites that expressed Tau at levels low enough to produce only a mild disruption of the structure of the ommatidia of the eye. This has a major advantage because, although the ratio of 4R:3R Tau varies between tauopathies (Ingelsson et al., 2007), Tau is not thought to be significantly overexpressed in AD. Thus, our model system, in which Tau is not highly overexpressed, may be more useful for uncovering mechanisms underlying Tau toxicity in disease.

Using site-specific integration, we examined the toxicity of two Tau isoforms (0N4R and 2N4R), with or without a point mutation, R406W, which is found in some autosomal dominant forms of FTLD-Tau (Hutton et al., 1998). Previous studies by others have reported a strong, highly disrupted eye phenotype when 0N4R R406W Tau is overexpressed in the *Drosophila* visual system, indicating enhanced toxicity (Wittmann et al., 2001; Jackson et al., 2002; Nishimura et al., 2004). We confirmed the increased toxicity of 0N4R Tau R406W *in vivo* using a previously generated strain with a randomly integrated Tau transgene (Wittmann et al., 2001). However, we found that, when we controlled for the integration site and reduced Tau overexpression using φC31-mediated site-specific integration, we failed to see any increase in toxicity caused by this mutation. We were also unable to detect any difference in toxicity generated by expression of the 0N4R and 2N4R Tau isoforms. Doubling the copy number of each of the UAS-transgenes increased the amount of toxicity observed, as expected from the increased expression of Tau. However, despite two copies of UAS-Tau increasing Tau expression to a level similar to that of the 0N4R Tau R406W line developed previously (Wittmann et al., 2001), the R406W mutation still had no effect on the organisation of the *Drosophila* eye. When we controlled for positional effects, our results suggest that the R406W mutation does not have a significant effect on Tau-mediated toxicity. Interestingly, this conclusion is in agreement with previous studies assaying the effect of FTLD-Tau-associated point mutations on the microtubule-binding properties of Tau (Delobel et al., 2002; Bunker et al., 2006). In an *in vitro* study using purified microtubules (Bunker et al., 2006) and an *in vivo* assay in Xenopus oocytes (Delobel et al., 2002), Tau R406W displayed only subtle differences in microtubule-binding compared to wild-type Tau. Taken together, these findings are consistent with the late onset of symptoms and slow disease progression observed in FTLD-Tau patients carrying the R406W Tau mutation (Heutink, 2000).

### GSK3β-mediated Tau toxicity is enhanced by S404A

GSK3β is a key candidate pathological Tau kinase in AD (Hanger et al., 1992; Lovestone et al., 1994; Lucas et al., 2001) to the extent that lithium and other GSK3β inhibitors have been trialled clinically for AD (reviewed by Mangialasche et al., 2010). GSK3β can phosphorylate many residues on Tau *in vitro* but it is not yet clear how each phosphorylation event contributes to Tau toxicity (Hanger et al., 2007) or whether all sites increase toxicity. We examined the role of priming kinases as a possible level of regulation. However, we were unable to detect any significant role for CK1δ or DYRK1A on Tau toxicity in this model system. Although hGSK3β did increase Tau toxicity, in our study it was not possible to identify a specific phosphorylation event that is responsible for this increased toxicity, suggesting that phosphorylation at multiple residues generate toxicity confirming previous observations investigating endogenous kinases (Steinhilb et al., 2007a; Steinhilb et al., 2007b; Chatterjee et al., 2009). Unexpectedly we found that phosphorylation of S404 in Tau appeared to be protective when co-expressed with hGSK3β, and substitution of S404 with alanine resulted in an enhanced toxicity compared to expressing either S404A or hGSK3β alone. A previous study examining the role of phosphorylation for Tau-mediated toxicity in the *Drosophila* eye identified that the double mutant S396A/S404A did not affect Tau toxicity (Steinhilb et al., 2007a) produced from endogenous kinases. We also found that S404A did not affect toxicity when acted on by endogenous kinases but see an enhancement of toxicity when S404A Tau was expressed together with hGSK3β together. We did not observe any phosphorylation of Ser396/Ser404 by endogenous *Drosophila* kinases but this epitope was phosphorylated when hGSK3β was co-expressed with Tau. Our results contrast therefore with the previous study, in which phosphorylation by endogenous kinases was detected at this site (Steinhilb et al., 2007a). This difference may be due to the increased toxicity of Tau in the previous study due to elevated Tau expression, which could potentially affect the recognition of Tau by endogenous kinases.

Importantly, although increased Tau phosphorylation is considered by many to be pathological, our observations indicate that some phosphorylation events may have a protective effect. Previous studies have also indicated that phosphorylation of Tau may reduce toxicity (Thies and Mandelkow, 2007). The PHF1 epitope (comprising pSer396 and pSer404) appears to be dynamically regulated by stress. Phosphorylation of these Tau residues is affected in various stress situations *in vivo*, including post-ischemic injury (Wen et al., 2004; Gordon-Krajcer et al., 2007) and following anaesthesia-induced hypothermia (Planel et al., 2007). *In vitro*, both phosphorylation (Gómez-Ramos et al., 2003) and dephosphorylation (Zambrano et al., 2004) of Ser396/Ser404 have been reported under conditions of oxidative stress. These data presumably reflect dynamic regulation: in ischaemic injury, Ser396/Ser404 becomes almost completely dephosphorylated, followed by a rapid increase in phosphorylation (Gordon-Krajcer et al., 2007). The phosphorylation of Ser396/Ser404 in neuroblastoma cells treated with lipid peroxidising agents can be blocked by inhibitors of either GSK3β or the stress-activated kinase p38, but not by cyclin-dependent kinase-5, supporting the view that phosphorylation of this epitope could be part of a co-ordinated neuronal stress response. It has been argued that increased Tau phosphorylation is a protective response to stress, rather than a primary cause of neuronal toxicity (Castellani et al., 2008; Buée et al., 2010), a view supported by evidence that expression of hypophosphorylated forms of Tau can also cause neurotoxicity (Chatterjee et al., 2009; Talmat-Amar et al., 2011). The inability to phosphorylate Ser404 combined with increased GSK3β activity may lead to a pattern of Tau phosphorylation that modifies its microtubule binding properties in such a way that toxicity is increased, potentially by generating a form that irreversibly binds microtubules. Alternatively GSK3β may modify additional components that contribute to Tau toxicity via a mechanism that is sensitive to the phosphorylation load on Tau. GSK3β expression can increase the toxicity of some but not all phosphorylation resistant forms of Tau, suggesting an interplay between Tau phosphorylation status and further kinase dependent components. Our modification of the *Drosophila* *in vivo* toxicity model may help to decipher the different roles of specific Tau phosphorylation events leading to changes in Tau toxicity.

## Materials and Methods

### *Drosophila* husbandry and stocks

Flies were maintained on standard cornmeal agar medium. Crosses were maintained at 18°C or 25°C on semi-defined rich “German food”. Medium recipes are available from the Bloomington Drosophila Stock Centre website. Transgenic flies were generated by standard P-element mediated transgenesis or φC31-mediated transgenesis by BestGene Inc. (Chino Hills, CA, USA) or GenetiVision (Houston, TX, USA). All transgenic flies produced were confirmed by sequencing and Splinkerette PCR was used to identify genomic DNA flanking regions (Potter and Luo, 2010). The control strain used for all experiments was an isogenic *w^1118^* line (Vienna Drosophila RNAi Center). Details of genetic markers and balancer chromosome are described at Flybase (http://flybase.org). GMR-GAL4 was used to drive expression of UAS-Tau and UAS-kinases in the visual system as previously described (Tuxworth et al., 2009).

### Molecular biology

#### Generation of UAS constructs

Full length open reading frames (ORFs) of cDNAs for Tau and each kinase were amplified with proof-reading Taq polymerase, cloned into pENTR (Invitrogen) and verified by sequencing. Constructs were recombined into the Murphy collection of Destination vectors supplied by the Drosophila Genomic Resource Centre (Bloomington, IN). The kinases were epitope-tagged with Myc or Flag sequences, while Tau was not tagged.

#### Site-directed mutagenesis of Tau

The ORF of 2N4R human Tau cloned into pTW was mutated using the QuikChange Multi kit (Stratagene) and confirmed by sequencing.

#### cDNA synthesis

RNA was extracted from 5–10 adult fly heads using Tri Reagent (Sigma) and used immediately for cDNA synthesis with the ImProm-II^TM^ Reverse Transcription System (Promega) following the manufacturer's instructions. 500 ng RNA was used per reaction.

#### qPCR

Quantitative PCR was performed by QStandard (http://www.qstandard.co.uk). Transcript levels for the following genes were quantified: *Drosophila* actin5c (CG4027), *Drosophila* GAPDH2 (CG8893), *Drosophila* EIF-4a (CG9075), mouse CD8a (geneID: 12525), human Tau (geneID: 4137).

### Imaging of fly eyes

#### 

Whole flies were processed for scanning electron microscopy or light microscopy and imaged as described (Tuxworth et al., 2009).

### Biochemistry

#### *Homogenisation of* Drosophila *heads*

Fly heads were used as material for qPCR and biochemistry. Whole flies were snap-frozen in liquid nitrogen and shaken at 6.5 m/s for 20 secs in a FastPrep24 homogeniser (MP Biomedical) to decapitate. Fly heads were separated from thoraces and abdomens by shaking through a fine sieve.

A two-stage, neutral-alkaline extraction procedure was used to maximise recovery of Tau protein. Fly heads were homogenised (20 heads/100 µl) in 100 µl ice-cold homogenisation buffer (50 mM Tris-HCl; 300 mM NaCl; 1% v/v β-mercaptoethanol, protease and phosphatase inhibitor cocktails [Calbiochem]), pH 6.8, and garnet beads in a FastPrep24 homogeniser (MP Biomedicals). Heads were homogenised twice at 6.5 m/s for 20 s and centrifuged at 20,000 g for 10 min at 4°C. The supernatant was removed and stored on ice. A second homogenisation was then performed by adding 100 µl of ice-cold homogenisation buffer at pH 9.2 to the pellet and centrifuging as above. The supernatants were combined, the pH adjusted to 8.0 and then centrifuged at 20,000 g for 30 min at 4°C. The final supernatant was stored at −80°C.

#### Sarcosyl-solubility of Tau

Ice-cold Tris buffer (10 mM Tris-HCl, pH 7.5, 800 mM NaCl, 1 mM EGTA, pH 8.0, 10% w/v sucrose, supplemented with protease and phosphatase inhibitor cocktails) was added to the fly heads (30 heads/60 µl buffer) and garnet beads. Fly heads were homogenised three times at 6.5 m/s for 20 s as above, then centrifuged at 1,000 g for 5 min at 4°C. The supernatant was removed and stored at −80°C and referred to as the sarcosyl-soluble fraction. N-lauroylsarcosinate (Sigma) was added to the pellet to give a final concentration of 1% (w/v) and the suspension was incubated for 1 h at ambient room temperature with gentle shaking. The suspension was centrifuged at 100,000 g for 1 h at 4°C. The insoluble pellet was resuspended in 50 mM Tris-HCl, pH 7.5, stored at −80°C and referred to as the sarcosyl-insoluble fraction.

#### Western blotting

SDS-PAGE, Western blotting, membrane blocking and probing were all performed by standard protocols. The membrane used was supported nitrocellulose (BioRad) which was blocked for 1 h in 5% milk in Tris-buffered saline (TBS) without Tween-20. Antibodies were diluted in 5% milk in TBS, 0.1% Tween-20 (TBST). Blots were washed with TBST, followed by two washes in TBS without Tween prior to imaging.

#### Antibodies used

Primary antibodies raised against: total human Tau (phosphoinsensitive), used at 1/10,000 (Dako); PHF1 human Tau pS396/pS404, used at 1/5,000 (gift of P. Davies, Albert Einstein College of Medicine, NY); AT8 human Tau pS202/pT205 at 1/2,000 (Innogenetics); Tau1 human Tau 197–208 at 1/1,000 (Chemicon); AT100 human Tau pT212/pS214 at 1/1,000 (Innogenetics); AT270 human Tau pT181 at 1/1,000 (Innogenetics); 9E10 c-Myc at 1/1,000 (Santa Cruz); M2 Flag-tag at 1/1,000 (Sigma); β-actin at 1/5,000 (Calbiochem). Secondary antibodies: Alexa-680 mouse anti-IgG at 1/15,000 (Invitrogen); Alexa-800 mouse anti-IgM at 1/15,000 (Rockland); Alexa-800 rabbit anti-IgG at 1/15,000 (Rockland).

#### Signal quantification

Membranes were scanned and quantified using an Odyssey infra-red scanner (Licor Biosciences) and Prism 5 software (GraphPad).

#### Normalisation of Tau levels between membranes

A constant amount of recombinant Tau was included in an individual lane on each Tau protein gel to act as a normalisation control.

### Statistical analysis

Statistical analysis was performed using Prism 5 (GraphPad). Transcript and protein quantification data were analysed for significance by one-way ANOVA with Bonferroni correction. Resulting p values lower than 0.05 were deemed significant.

## Supplementary Material

Supplementary Material
