# Supplementary material for: Modification of the Drosophila model of in vivo Tau toxicity reveals protective phosphorylation by GSK3β
Source: Biol Open. 2013 Nov 19;3(1):1–11. doi: 10.1242/bio.20136692 (PMC3892155; doi:10.1242/bio.20136692)
Supplement: Supplementary Material [file supp_3_1_1_v2_index.html]

Modification of the Drosophila model of in vivo Tau toxicity reveals protective phosphorylation by GSK3β — Supplementary Material 

# Modification of the *Drosophila* model of *in vivo* Tau toxicity reveals protective phosphorylation by GSK3β

## bio.20136692 Supplementary Material

**Files in this Data Supplement:**

- Supplementary Material - Giulia Povellato et al. doi: 10.1242/bio.20136692
