# Supplementary material for: Modification of the Drosophila model of in vivo Tau toxicity reveals protective phosphorylation by GSK3β
Source: Biol Open. 2013 Nov 19;3(1):1–11. doi: 10.1242/bio.20136692 (PMC3892155; doi:10.1242/bio.20136692)
Supplement: Supplementary Material [file supp_bio.20136692_bio.20136692-s1.pdf]

Supplementary Material

Giulia Povellato et al. doi: 10.1242/bio.20136692

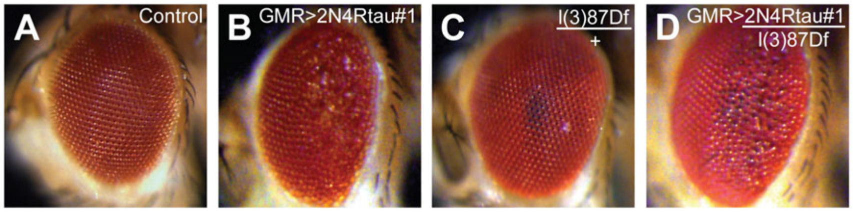

Fig. S1. Light micrographs of eyes from 0–5 day old flies. (A) Control eyes displaying the regular array of ommatidia on the external surface of the compound eye. (B) Disruption of the ommatidial structure when Tau is overexpressed from insertion line 1. (C) Flies heterozygous for l(3)87Df do not exhibit ommatidial disruption. (D) The human Tau-mediated eye phenotype in 2N4R Tau #1 is not enhanced by heterozygote mutations in l(3)87Df. Genotypes are: A: GMR-gal4/+ ; +/+, B: GMR-gal4/+ ; UAS-2N4R Tau #1/+, C: +/+ ; l(3)87Df/+, D: GMR-gal4/+ ; UAS-2N4R Tau #1/l(3)87Df.

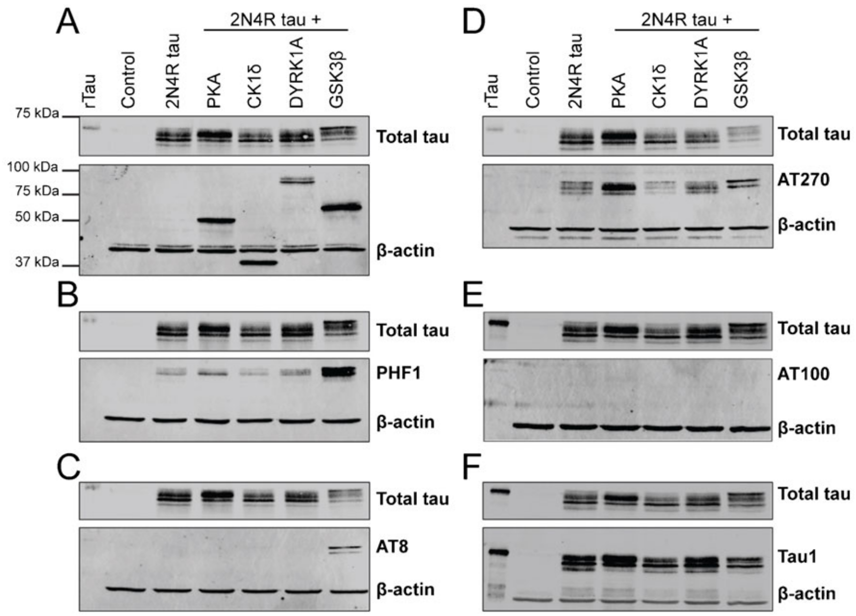

Fig. S2. Human GSK3β phosphorylates human Tau in *Drosophila* cells. *Drosophila* S2 cells were transiently transfected with constructs to express human 2N4R Tau alone or with one of human PKA subunit α, CK1δΔ317, GSK3β or rat DYRK1A. The kinases were epitope-tagged with Flag or Myc sequences. Cell lysates were Western blotted with recombinant human 2N4R Tau included on each gel as a control. (A) Multiple Tau bands at 60–70 kDa are detected with a phospho-independent anti-Tau (Dako). PKA (50 kDa), CK1δ (37 kDa), DYRK1A (95 kDa), GSK3β (60 kDa) were detected at the expected sizes. Phosphorylated human Tau was detected using phospho-specific antibodies (B) PHF1 for pS396/pS404, (C) AT8 for pS202/pT205 and (D) AT270 for pT181. (E) No pT212/pS214 Tau is detectable using AT100. (F) Tau1 detects dephosphosphorylated Tau1 S198–S208. Human 2N4R tau expressed alone shows a basal phosphorylation level at the PHF1 and AT270 epitopes. Co-transfection of hGSK3β causes a mobility shift of human Tau indicating increased phosphorylation load. Increased phosphorylation is also detected with PHF1, AT8 and AT270 antisera.

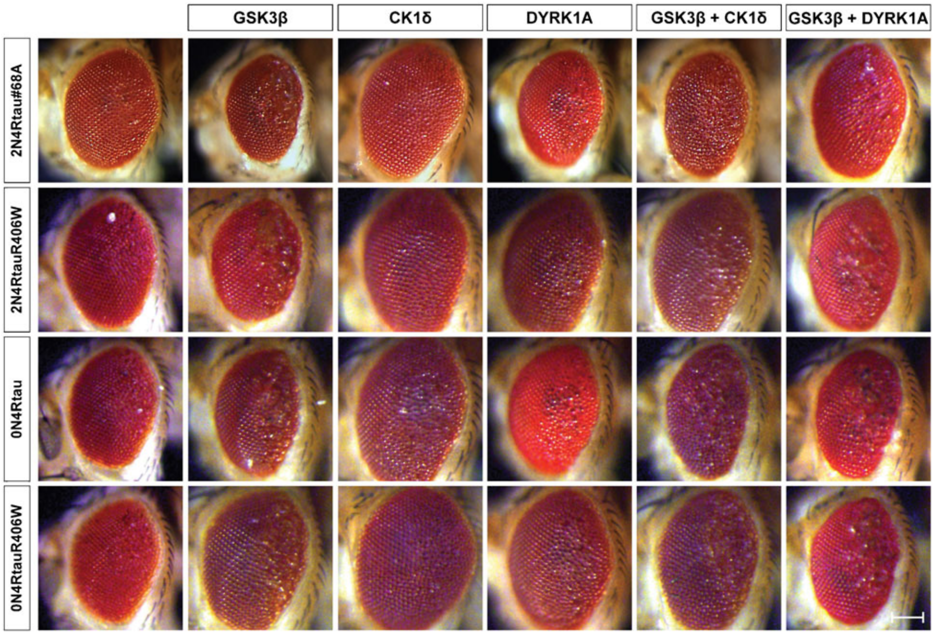

Fig. S3. Priming kinases CK1δ and DYRK1A do not affect the increase in tau toxicity mediated by GSK3β. Human 2N4R or 0NR Tau with or without the R406W mutation were expressed from the 68A locus under the control of GMR-gal4. hGSK3β, human CK1δΔ317 or rat DYRK1A were co-expressed with the tau isoforms and the effect on toxicity determined. Co-expression of hGSK3β increases the toxicity of each tau isoform but addition of CK1δ or DYRK1A did not further increase toxicity.

Table S1. Genomic insertion sites for UAS-2N4R tau lines generated by random insertion transgenesis. The chromosomal locations of the insertions were determined by splinkerette PCR. The closest genes and their predicted functions are based on Flybase annotations.

Line	Chromosome	Nearest gene	CG no.	Likely function
1	3L	neurotactin	9704	Cell adhesion
2	2L	CG17341	17341	Unknown protein
3	3R	l(3)87Df	7620	Cytochrome oxidase biogenesis
4	3R	CG7381	7381	EB domain, EGF-domain
5	2R	MrgBP	13746	Chromatin modification
